# Water-Induced Regeneration of a 2,2-Diphenyl-1-picrylhydrazyl Radical after Its Scandium Ion-Promoted Electron-Transfer Disproportionation in an Aprotic Medium

**DOI:** 10.3390/molecules28135002

**Published:** 2023-06-26

**Authors:** Ikuo Nakanishi, Yoshimi Shoji, Kei Ohkubo, Hiromu Ito, Shunichi Fukuzumi

**Affiliations:** 1 Quantum RedOx Chemistry Team, Institute for Quantum Life Science (iQLS), Quantum Life and Medical Science Directorate, National Institutes for Quantum Science and Technology (QST), Inage-ku, Chiba 263-8555, Japan; shoji.yoshimi@qst.go.jp (Y.S.); ito.hiromu@qst.go.jp (H.I.); 2 Institute for Advanced Co-Creation Studies, Open and Transdisciplinary Research Initiatives, Osaka University, Suita 565-0871, Japan; 3 Department of Chemistry and Nano Science, Ewha Womans University, Seoul 03760, Republic of Korea; 4 Department of Chemistry, Faculty of Pure and Applied Sciences, University of Tsukuba, Tsukuba 305-8571, Japan

**Keywords:** radical, electron transfer, disproportionation, scandium ion, Lewis acid, comproportionation, EPR, kinetics, reaction mechanism

## Abstract

A neutral, stable radical, 2,2-diphenyl-1-picrylhydrazyl radical (DPPH^•^), has been frequently used to estimate the activity of antioxidants for more than 60 years. However, the number of reports about the effect of metal ions on the reactivity of DPPH^•^ is quite limited. We have recently reported a unique electron-transfer disproportionation of DPPH^•^ to produce the DPPH cations (DPPH^+^) and anions (DPPH^−^) upon the addition of scandium triflate [Sc(OTf)_3_ (OTf = OSO_2_CF_3_)] to an acetonitrile (MeCN) solution of DPPH^•^. The driving force of this reaction is suggested to be an interaction between DPPH^–^ and Sc^3+^. In this study, it is demonstrated that the addition of H_2_O to the DPPH^•^–Sc(OTf)_3_ system in MeCN resulted in an increase in the absorption band at 519 nm due to DPPH^•^. This indicated that an electron-transfer comproportionation occurred to regenerate DPPH^•^. The regeneration of DPPH^•^ was also confirmed by electron paramagnetic resonance (EPR) spectroscopy. The amount of DPPH^•^ increased with an increasing amount of added H_2_O to reach a constant value. The detailed mechanism of regeneration of DPPH^•^ was proposed based on the detailed spectroscopic and kinetic analyses, in which the reaction of DPPH^+^ with [(DPPH)_2_Sc(H_2_O)_3_]^+^ generated upon the addition of H_2_O to [(DPPH)_2_Sc]^+^ is the rate-determining step.

## 1. Introduction

**Figure 1:**
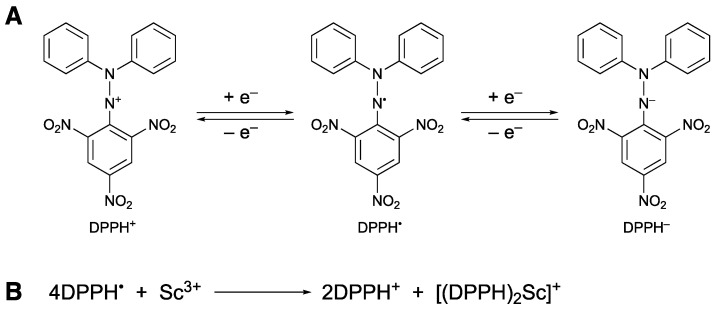
(**A**) Redox behavior or DPPH^•^. (**B**) Sc^3+^-induced disproportionation of DPPH^•^.

2,2-Diphenyl-1-picrylhydrazyl radical (DPPH^•^) is a neutral, stable radical that has been frequently used to estimate the activity of antioxidants for more than 60 years [1,2,3,4]. It is known that the radical-scavenging reactivity of antioxidants is significantly affected by the reaction environments, such as solvents [5,6], pH [7,8], the presence of metal ions [9,10,11,12,13,14,15,16,17,18], and so on. However, the number of reports about the reactivity of DPPH^•^ in the presence of metal ions is quite limited. We have demonstrated that the DPPH^•^-scavenging reactivity of phenolic compounds, such as a vitamin E model, flavonoids, and hydroquinones, is significantly enhanced in the presence of redox-inactive metal ions with a moderate Lewis acidity, such as Mg^2+^ [10] and Al^3+^ [9]. The coordination of the metal ion to the one-electron reduced species of DPPH^•^ (DPPH^–^) may stabilize the product, resulting in the acceleration of the electron transfer. On the other hand, DPPH^•^ is known to undergo reversible one-electron reduction and oxidation to produce DPPH^−^ and the corresponding cation (DPPH^+^), respectively, in organic solvents (Figure 1A) [19,20,21,22]. We have also reported that an electron-transfer disproportionation of DPPH^•^ to produce DPPH^+^ and DPPH^–^ occurs upon the addition of scandium triflate [Sc(OTf)_3_ (OTf = OSO_2_CF_3_)] to an acetonitrile (MeCN) solution of DPPH^•^ [23]. Since there is no proton sauce in this reaction system, DPPH^−^ does not undergo protonation to produce DPPH-H. Then, DPPH^−^ may significantly be stabilized by the strong Lewis acidity of Sc^3+^ with a formation constant of 2.3 × 10^3^ M^–1^. Recently, Denzo et al. have reported the reactivity of DPPH^•^ in the presence of metal cations (Cu^2+^ and Zn^2+^) and acids (HClO_4_ and HNO_3_) in MeCN [24]. A strong Brønsted acid, such as HClO_4_, is required for the disproportionation of DPPH^•^ to occur. We report herein that the addition of water to the MeCN solution containing DPPH^+^, DPPH^−^ and Sc(OTf)_3_ resulted in the electron-transfer comproportionation between DPPH^+^ and DPPH^–^ to regenerate DPPH^•^, demonstrating the reversibility of the Sc^3+^-catalyzed electron-transfer disproportionation of DPPH^•^. The reversible redox reactivity of DPPH^•^ in the presence of the redox-inactive metal ion with strong Lewis acidity shows a unique electron-transfer redox reaction of radical species in aprotic media.

## 2. Results and Discussion

**Figure 2:**
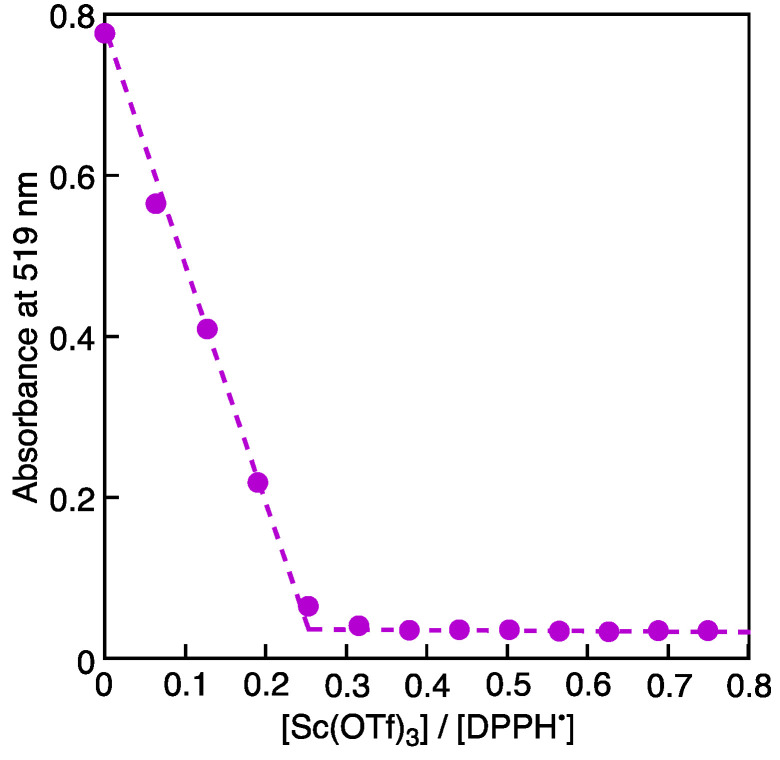
Plot of the absorbance at 519 nm vs. [Sc(OTf)_3_]/[DPPH^•^] in MeCN. [DPPH^•^] and [Sc(OTf)_3_] are concentrations of DPPH^•^ (6.6 × 10^–5^ M) and Sc(OTf)_3_ (4.2 × 10^–6^ M, each), respectively.

When Sc(OTf)_3_ was added to an MeCN solution of DPPH^•^, a decrease in the absorption band of DPPH^•^ at 519 nm was observed, accompanied by an increase in the absorption band at 380 nm due to the electron-transfer disproportionation [23]. The band at 380 nm is characteristic of DPPH^+^. The spectral titration conducted in this study shows the Sc(OTf)_3_/DPPH^•^ molar ratio being 1:4 (Figure 2). Thus, two molecules of DPPH^–^ are suggested to be stabilized by one Sc^3+^, as shown in Figure 1B, although the [(DPPH)_2_Sc]^+^ complex has yet to be detected.

**Figure 3:**
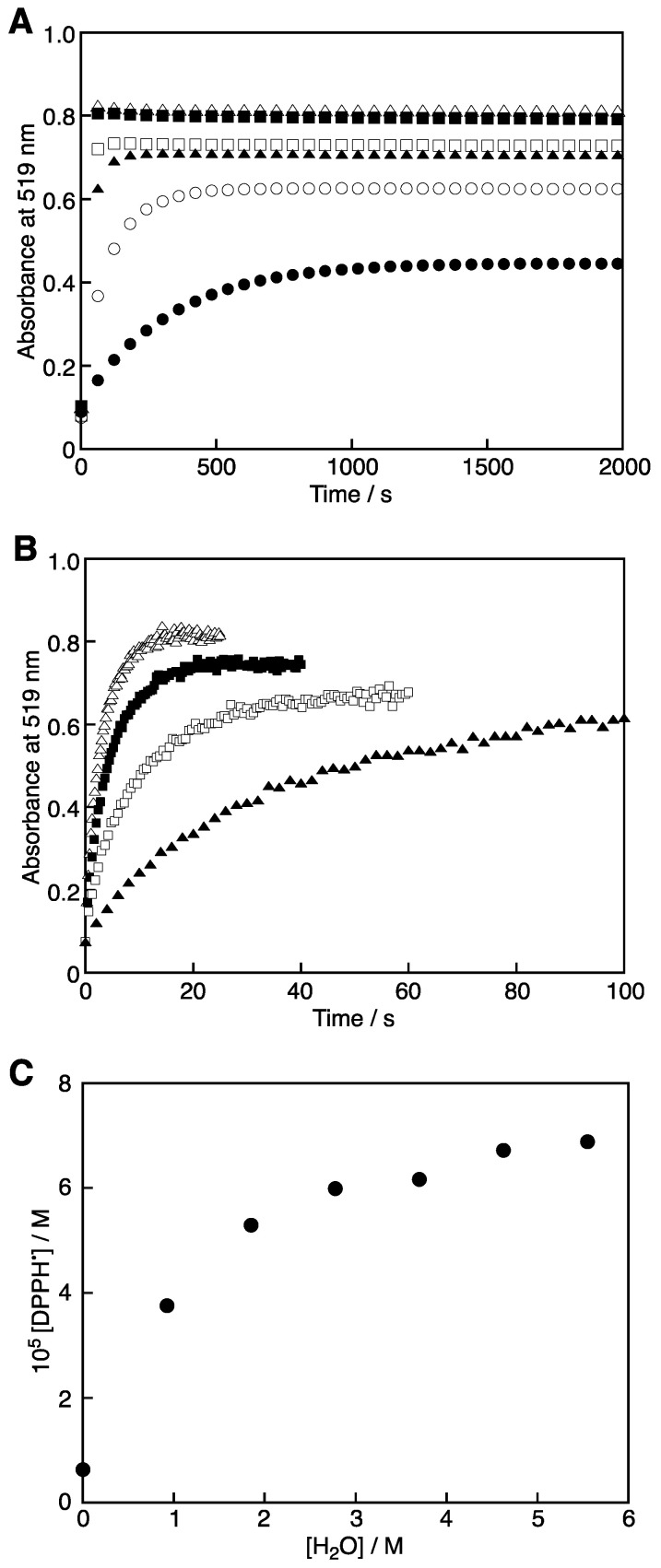
(**A**) Time course change monitored by the Agilent 8453 photodiode array spectrophotometer in the absorbance at 519 nm after the addition of H_2_O (closed circles 50 µL (9.3 × 10^−1^ M), open circles 100 µL (1.9 M), closed triangles 150 µL (2.8 M), open squares 200 µL (3.7 M), closed squares 250 µL (4.2 M), and open triangles 300 µL (5.6 M)) to the MeCN solution containing DPPH^•^ and Sc(OTf)_3_ at 298 K. The final concentrations of DPPH^•^ and Sc(OTf)_3_ are 7.1 × 10^−5^ M and 2.0 × 10^−5^ M, respectively, in 3 mL MeCN–H_2_O. (**B**) Time course change monitored by the stopped-flow spectrophotometer in the absorbance at 519 nm after addition of H_2_O (closed triangles 150 µL (2.8 M), open squares 200 µL (3.7 M), closed squares 250 µL (4.2 M), and open triangles 300 µL (5.6 M)) to the MeCN solution containing DPPH^•^ and Sc(OTf)_3_ at 298 K. The final concentrations of DPPH^•^ and Sc(OTf)_3_ are 7.1 × 10^–5^ M and 2.0 × 10^–5^ M, respectively, in 3 mL MeCN–H_2_O. (**C**) Plot of [DPPH^•^] vs. [H_2_O] at 1500 s after the addition of H_2_O to the MeCN solution containing DPPH^•^ and Sc(OTf)_3_. The final concentrations of DPPH^•^ and Sc(OTf)_3_ are 7.1 × 10^–5^ M and 2.0 × 10^–5^ M, respectively, in 3 mL MeCN–H_2_O.

**Figure 4:**
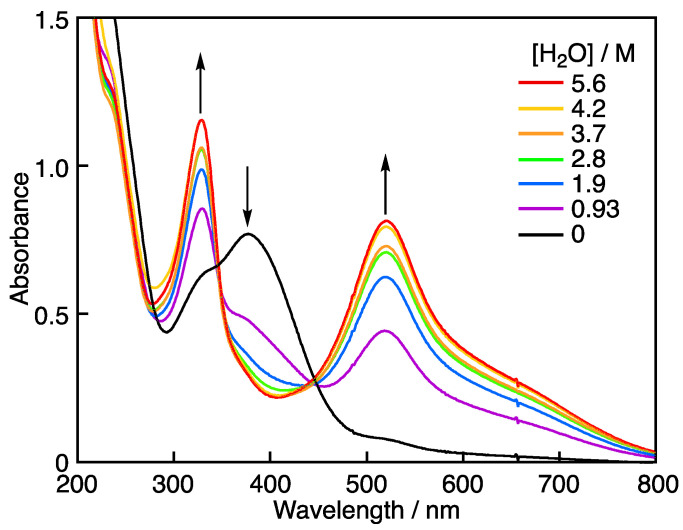
Spectral change observed 1500 s after the addition of H_2_O (9.3 × 10^–1^, 1.9, 2.8, 3.7, 4.2, and 5.6 M) to the MeCN solution containing DPPH^•^ and Sc(OTf)_3_. The final concentrations of DPPH^•^ and Sc(OTf)_3_ are 7.1 × 10^–5^ M and 2.0 × 10^–5^ M, respectively, in 3 mL MeCN–H_2_O.

Upon the addition of H_2_O to this solution, the absorption band at 519 nm due to DPPH^•^ increased. The time course changes in the absorbance at 519 nm after the addition of several amounts of H_2_O are shown in Figure 3A,B. At all the concentrations of H_2_O, the reaction has completed after 1500 s. Figure 4 shows the overlapped absorption spectra at 1500 s after the addition of varying amounts of H_2_O. The absorption band at 380 nm due to DPPH^+^ decreased, accompanied by an increase in the absorption band at 519 nm due to DPPH^•^ with clear isosbestic points at 344 and 449 nm.

**Figure 5:**
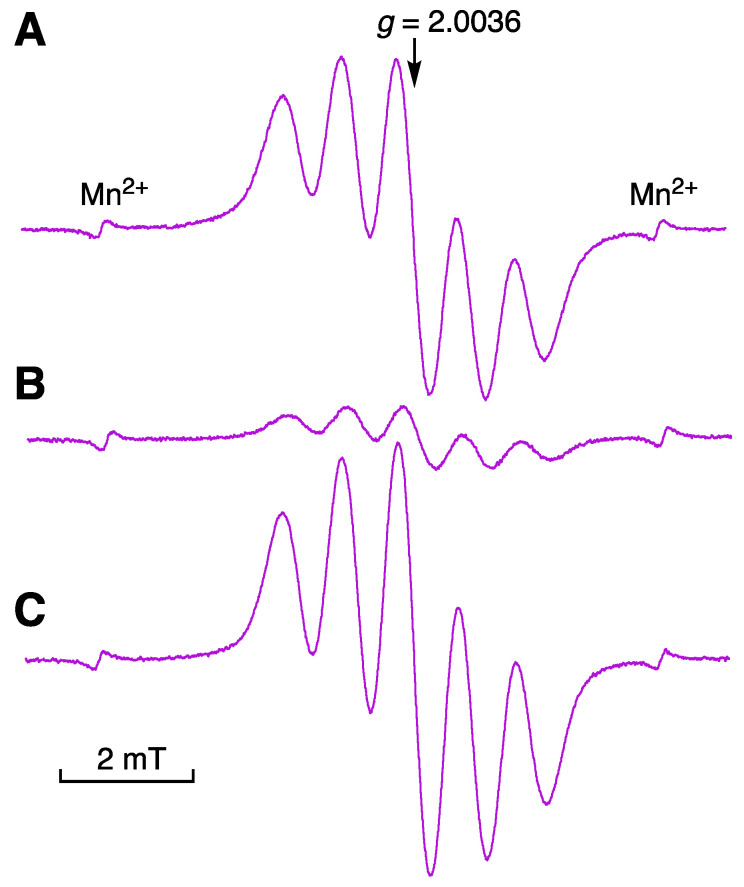
EPR spectra of (**A**) DPPH^•^ (7.1 × 10^−5^ M) in MeCN, (**B**) DPPH^•^ (7.1 × 10^−5^ M) in the presence of Sc(OTf)_3_ (2.0 × 10^−5^ M) in MeCN, and (**C**) DPPH^•^ (7.1 × 10^−5^ M)in the presence of Sc(OTf)_3_ (1.8 × 10^−5^ M) and H_2_O (5.6 M) in MeCN.

The regeneration of DPPH^•^ upon the addition of H_2_O to the DPPH^•^–Sc(OTf)_3_ system in MeCN was also confirmed by the electron paramagnetic resonance (EPR) spectroscopy. The well-resolved five lines having a *g* value of 2.0036 were observed in the EPR spectrum of DPPH^•^ in MeCN (Figure 5A). Upon addition of Sc(OTf)_3_ to the MeCN solution of DPPH^•^, the signal intensity was significantly decreased, as shown in Figure 5B. The addition of H_2_O to this reaction system resulted in the regeneration of DPPH^•^, which was confirmed by the increase in the EPR signal intensity due to DPPH^•^ (Figure 5C).

Figure 3C shows [DPPH^•^] vs. [H_2_O] at 1500 s after the addition of H_2_O to the 3 mL MeCN solution containing DPPH^•^ (7.1 × 10^−5^ M) and Sc(OTf)_3_ (2.0 × 10^−5^ M). The [DPPH^•^] values were calculated using the extinction coefficient (*ε*) of 1.2 × 10^4^ M^−1^ cm^−1^ at 519 nm [2] and increased with increasing [H_2_O] to reach a constant value. It is suggested that the complex formation of Sc^3+^ with H_2_O may weaken the interaction between DPPH^−^ and Sc^3+^, leading to the electron-transfer comproportionation to produce DPPH^•^. In fact, a hexaaqua complex, Sc(H_2_O)_6_^3+^, has been reported for the Sc^3+^ hydration in aqueous perchlorate solution [25].

**Figure 6:**
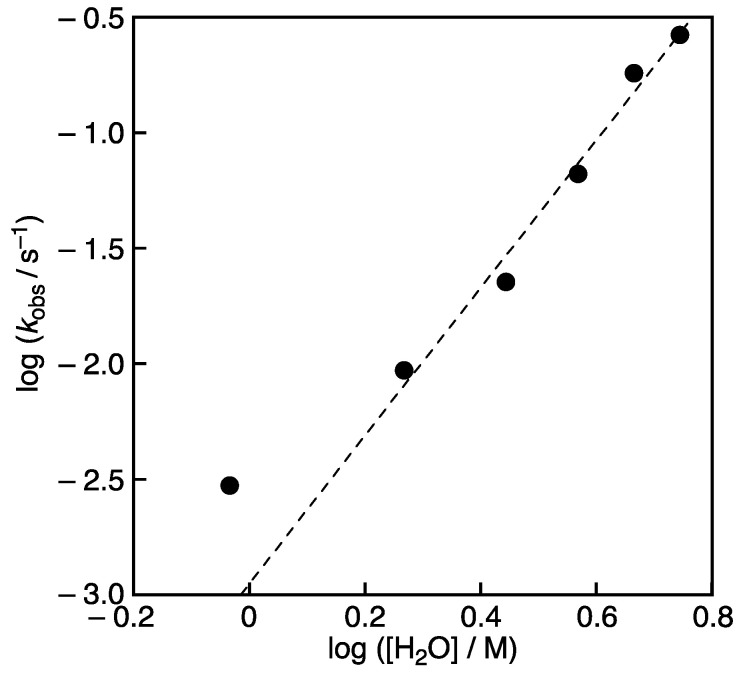
Plot of log *k*_obs_ vs. log[H_2_O].

**Figure 7:**
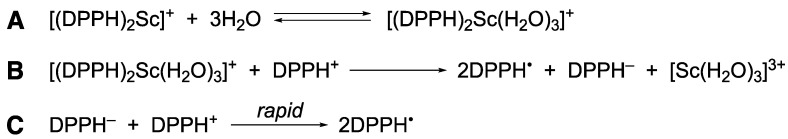
Proposed mechanism of the DPPH^•^ regeneration. (**A**) Addition of H_2_O to form [(DPPH)_2_Sc(H_2_O)_3_]^+^. (**B**) Rate-determining electron transfer from [(DPPH)_2_Sc(H_2_O)_3_]^+^ to DPPH^+^. (**C**) Rapid comproportionation between DPPH^–^ and DPPH^+^.

The rise of the absorbance at 519 nm due to DPPH^•^ shown in Figure 3A,B obeyed pseudo-first-order kinetics. Figure 6 shows a double logarithmic plot of the pseudo-first-order rate constants (*k*_obs_) vs. [H_2_O]. The slope of this plot (dashed line in Figure 6), except for the *k*_obs_ value at 9.3 × 10^−1^ M H_2_O, is about three, suggesting that a triaqua complex, [(DPPH)_2_Sc(H_2_O)_3_]^+^, may be formed by the addition of H_2_O to [Sc(DPPH)_2_]^+^ as shown in Figure 7A. Then, the reaction B (Figure 7) occurs to produce two molecules of DPPH^•^, DPPH^−^, and [Sc(H_2_O)_3_] as the rate-determining step followed by a rapid reaction between DPPH^−^ and DPPH^+^ to produce two molecules of DPPH^•^ (Figure 7C).

## 3. Materials and Methods

### 3.1. Materials

DPPH^•^ was commercially obtained from Tokyo Chemical Industry Co., Ltd., Tokyo, Japan. Sc(OTf)_3_ was purchased from Sigma–Aldrich, St. Louis, MO, USA. MeCN (spectral grade) used as a solvent was commercially obtained from Nacalai Tesque, Inc., Kyoto, Japan, and used as received. The water used in this study was freshly prepared with a Milli-Q system (Millipore Direct-Q UV3) (Merck Millipore, Burlington, MA, USA).

### 3.2. Spectral Measurements

Typically, a 10 µL aliquot of Sc(OTf)_3_ (6.1 × 10^−3^ M) in MeCN was added to a quartz cuvette (10 mm i.d.) which contained DPPH^•^ in MeCN (2.95, 2.90, 2.85, 2.80, 2.75 or 2.70 mL). This led to an electron-transfer disproportionation of DPPH^•^ to produce DPPH^+^ and [(DPPH)_2_Sc]^+^. After 1 h, water (50, 100, 150, 200, 250, or 300 µL) was added to this MeCN solution (2.95, 2.90, 2.85, 2.80, 2.75, or 2.70 mL, respectively). The molar concentrations of 50, 100, 150, 200, 250, and 300 µL H_2_O in 3 mL MeCN–H_2_O are 9.3 × 10^−1^, 1.9, 2.8, 3.7, 4.2, 5.6 M, respectively. The final concentrations of DPPH^•^ and Sc(OTf)_3_ were 7.1 × 10^−5^ M and 2.0 × 10^−5^ M, respectively, in 3 mL MeCN–H_2_O. UV-vis spectral changes associated with the reaction were monitored using an Agilent 8453 photodiode array spectrophotometer thermostated with a Peltier temperature control at 298 K (Agilent Technologies, Santa Clara, CA, USA). The regeneration rates of DPPH^•^ were followed by monitoring the absorbance change at 519 nm due to DPPH^•^ on the Agilent 8453 photodiode array spectrophotometer ([H_2_O] = 9.3 × 10^−1^ and 1.9 M) or on a UNISOKU RSP-1000-02NM stopped-flow spectrophotometer (UNISOKU Co., Ltd., Osaka, Japan), which was thermostated with a Thermo Scientific NESLAB RTE-7 Circulating Bath (Thermo Fisher Scientific, Inc., Waltham, MA, USA) at 298 K ([H_2_O] = 2.8, 3.7, 4.2, and 5.6 M). The *k*_obs_ values were obtained by a least-square curve fit using an Apple MacBook Pro personal computer (Apple Inc., Cupertino, CA, USA). The first-order plots of ln(*A*_∞_–*A*) vs. time (*A* and *A*_∞_ are the absorbance at the reaction time and the final absorbance, respectively) were linear until three or more half-lives, with a correlation coefficient ρ > 0.999. In each case, it was confirmed that the *k*_obs_ values derived from at least three independent measurements agreed within experimental error of ±5%. In all cases, solutions were normally equilibrated with air.

### 3.3. EPR Measurements

The EPR spectra of DPPH^•^ (7.1 × 10^−5^ M) in the presence or absence of Sc(OTf)_3_ (2.0 × 10^−5^ M) and/or H_2_O (5.6 M) in MeCN were taken using an LLC-04B ESR sample tube (LABOTEC Co., Ltd., Tokyo, Japan) on a JEOL X-band spectrometer (JES-RE1X) (JEOL Ltd., Tokyo, Japan) at room temperature under the following conditions: microwave frequency 9.43 GHz, microwave power 8 mW, center field 338 mT, sweep width 15 mT, sweep rate 3 mT min^−1^, modulation frequency 100 kHz, modulation amplitude 0.2 mT, and time constant 0.1 s. EPR data acquisition was controlled by the WIN-RAD ESR Sata Analyzer System (Radical Research, Inc., Tokyo, Japan). The *g* values were calibrated with an Mn^2+^ marker. In all cases, solutions were normally equilibrated with air.

## 4. Conclusions

The addition of H_2_O to the MeCN solution containing DPPH^+^ and [(DPPH)_2_Sc]^+^ resulted in the regeneration of DPPH^•^. It is suggested that the complex formation of Sc^3+^ with H_2_O may weaken the interaction between DPPH^−^ and Sc^3+^, leading to the electron-transfer comproportionation to produce DPPH^•^. The detailed mechanism of regeneration of DPPH^•^ was proposed based on the detailed spectroscopic and kinetic analyses, in which the reaction of DPPH^+^ with [(DPPH)_2_Sc(H_2_O)_3_]^+^ generated upon the addition of H_2_O to [(DPPH)_2_Sc]^+^ is the rate-determining step.

## Data Availability

Data is contained within the article.

## References

[1] Angeli L., Imperiale S., Ding Y., Scampicchio M., Morozova K. (2021). A novel stoichio-kinetic model for the DPPH• assay: The importance of the side reaction and application to complex mixtures. Antioxidants.

[2] Foti M.C. (2015). Use and abuse of the DPPH^•^ radical. J. Agric. Food Chem..

[3] Pyrzynska K., Pękal A. (2013). Application of free radical diphenylpicrylhydrazyl (DPPH) to estimate the antioxidant capacity of food samples. Anal. Methods.

[4] Blois M.S. (1958). Antioxidant determinations by the use of a stable free radical. Nature.

[5] Litwinienko G., Ingold K.U. (2007). Solvent effects on the rates and mechanisms of reaction of phenols with free radicals. Acc. Chem. Res..

[6] Valgimigli L., Banks J.T., Lusztyk J., Ingold K.U. (1999). Solvent effects on the antioxidant activity of vitamin E. J. Org. Chem..

[7] Mukai K., Tokunaga A., Itoh S., Kanesaki Y., Ohara K., Nagaoka S., Abe K. (2007). Structure–activity relationship of the free-radical-scavenging reaction by vitamin E (α-, β-, γ-, δ-tocopherols) and ubiquinol-10: pH dependence of the reaction rates. J. Phys. Chem. B.

[8] Mitani S., Ouchi A., Watanabe E., Kanesaki Y., Nagaoka S., Mukai K. (2008). Stopped-flow kinetic study of the aroxyl radical-scavenging action of catechins and vitamin C in ethanol and micellar solutions. J. Agric. Food Chem..

[9] Nakanishi I., Ohkubo K., Ogawa Y., Matsumoto K., Ozawa T., Fukuzumi S. (2016). Aluminium ion-promoted radical-scavenging reaction of methylated hydroquinone derivatives. Org. Biomol. Chem..

[10] Waki T., Kobayashi S., Ozawa T., Kamada T., Nakanishi I. (2013). Effects of ionic radius of redox-inactive bio-related metal ions on the radical-scavenging activity of flavonoids evaluated using photometric titration. Chem. Commun..

[11] Nakanishi I., Shimada T., Ohkubo K., Manda S., Shimizu T., Urano S., Okuda H., Miyata N., Ozawa T., Anzai K. (2007). Involvement of electron transfer in the radical-scavenging reaction of resveratrol. Chem. Lett..

[12] Nakanishi I., Kawaguchi K., Ohkubo K., Kawashima T., Manda S., Kanazawa H., Takeshita K., Anzai K., Ozawa T., Fukuzumi S. (2007). Scandium-ion accelerated scavenging reaction of cumylperoxyl radical by a cyclic nitroxyl radical via electron transfer. Chem. Lett..

[13] Nakanishi I., Kawashima T., Ohkubo K., Kanazawa H., Inami K., Mochizuki M., Fukuhara K., Okuda H., Ozawa T., Itoh S. (2005). Electron-transfer mechanism in radical-scavenging reactions by a vitamin E model in a protic medium. Org. Biomol. Chem..

[14] Nakanishi I., Ohkubo K., Miyazaki K., Hakamata W., Urano S., Ozawa T., Okuda H., Fukuzumi S., Ikota N., Fukuhara K. (2004). A planar catechin analogue having a more negative oxidation potential than (+)-catechin as an electron transfer antioxidant against a peroxyl radical. Chem. Res. Toxicol..

[15] Nakanishi I., Miyazaki K., Shimada T., Ohkubo K., Urano S., Ikota N., Ozawa T., Fukuzumi S., Fukuhara K. (2002). Effects of metal ions distinguishing between one-step hydrogen- and electron-transfer mechanisms for the radical-scavenging reaction of (+)-catechin. J. Phys. Chem. A.

[16] Mukai K., Oi M., Ouchi A., Nagaoka S. (2012). Kinetic study of the α-tocopherol-regeneration reaction of ubiquinol-10 in methanol and acetonitrile solutions: Notable effect of the alkali and alkaline earth metal salts on the reaction rates. J. Phys. Chem. B.

[17] Kohno Y., Fujii M., Matsuoka C., Hashimoto H., Ouchi A., Nagaoka S., Mukai K. (2011). Notable effects of the metal salts on the formation and decay reactions of α-tocopheroxyl radical in acetonitrile solution. The complex formation between α-tocopheroxyl and metal cations. J. Phys. Chem. B.

[18] Ouchi A., Nagaoka S., Abe K., Mukai K. (2009). Kinetic study of the aroxyl radical-scavenging reaction of α-tocopherol in methanol solution: Notable effect of the alkali and alkaline earth metal salts on the reaction rates. J. Phys. Chem. B.

[19] Solon E., Bard A.J. (1964). The electrochemistry of diphenylpicrylhydrazyl. J. Am. Chem. Soc..

[20] Kalinowski M.K., Kimkiewicz J. (1983). Solvation effects in the electrochemistry of diphenylpicrylhydrazyl. Monatsh. Chem..

[21] Deutchoua A.D.D., Siegnin R., Kouteu G.K., Denzo G.K., Ngameni E. (2019). Electrochemistry of 2,2-diphenyl-1-picrylhydrazyl (DPPH) in acetonitrile in presence of ascorbic acid—Application for antioxidant properties evaluation. ChemistrySelect.

[22] Patrascu P., Lete C., Popescu C., Matache M., Paun A., Madalan A., Ionita P. (2020). Synthesis and spectral comparison of electronic and molecular properties of some hydrazines and hydrazyl free radicals. Arkivoc.

[23] Nakanishi I., Kawashima T., Ohkubo K., Waki T., Uto Y., Kamada T., Ozawa T., Matsumoto K., Fukuzumi S. (2014). Disproportionation of a 2,2-diphenyl-1-picrylhydrazyl as a model of reactive oxygen species catalysed by Lewis and/or Brønsted acids. Chem. Commun..

[24] Ngueumaleu Y., Deuchoua A.D.D., Hanga S.S.P., Liendji R.W., Denzo G.K., Ngameni E. (2023). Probing the reactivity of 2,2-diphenyl-1-picrylhydrazyl (DPPH) with metal cations and acids in acetonitrile by electrochemistry and UV-Vis spectroscopy. Phys. Chem. Chem. Phys..

[25] Rudolph W.W., Pye C.C. (2000). Raman spectroscopic measurements of scandium(III) hydration in aqueous perchlorate solution and ab intio molecular orbital studies of scandium(III) water clusters: Does Sc(III) occur as a hexaaqua complex?. J. Phys. Chem. A.

